# Up-Regulation of hsa-miR-210 Promotes Venous Metastasis and Predicts Poor Prognosis in Hepatocellular Carcinoma

**DOI:** 10.3389/fonc.2018.00569

**Published:** 2018-12-03

**Authors:** Jia Ji, Yuan Rong, Chang-Liang Luo, Shuo Li, Xiang Jiang, Hong Weng, Hao Chen, Wu-Wen Zhang, Wen Xie, Fu-Bing Wang

**Affiliations:** ^1^Department of Laboratory Medicine, Zhongnan Hospital of Wuhan University, Wuhan, China; ^2^Department of Laboratory Medicine, Wuhan Children's Hospital, Huazhong University of Science and Technology, Wuhan, China; ^3^Department of Hepatobiliary and Pancreatic Surgery, Zhongnan Hospital of Wuhan University, Wuhan, China; ^4^Center for Evidence-Based and Translational Medicine, Zhongnan Hospital of Wuhan University, Wuhan, China; ^5^Department of Pathology, Zhongnan Hospital of Wuhan University, Wuhan, China

**Keywords:** hsa-miR-210, venous metastasis, prognosis, hepatocellular carcinoma, bioinformatics analysis, RT-qPCR, public gene database

## Abstract

**Objective:** To investigate the potential biomarkers for venous metastasis of hepatocellular carcinoma (HCC), and briefly discuss their target genes and the signaling pathways they are involved in.

**Materials and Method:** The dataset GSE6857 was downloaded from GEO. Significantly differentially expressed miRNAs were identified using the R package “limma,” After that, the survival analysis was conducted to discover the significance of these up-regulated miRNAs for the prognosis of HCC patients. Additionally, miRNAs which were up-regulated in venous metastasis positive HCC tissues and were significant for the prognosis of HCC patients were further verified in clinical samples using RT-qPCR. The miRNAs were then analyzed for their correlations with clinical characteristics including survival time, AFP level, pathological grade, TNM stage, tumor stage, lymph-node metastasis, distant metastasis, child-pugh score, vascular invasion, liver fibrosis and race using 375 HCC samples downloaded from the TCGA database. The target genes of these miRNAs were obtained using a miRNA target gene prediction database, and their functions were analyzed using the online tool DAVID.

**Results:** 15 miRNAs were differentially expressed in samples with venous metastasis, among which 7 were up-regulated in venous metastasis positive HCC samples. As one of the up-regulated miRNAs, hsa-miR-210 was identified as an independent prognostic factor for HCC. Using RT-qPCR, it was evident that hsa-miR-210 expression was significantly higher in venous metastasis positive HCC samples (*p* = 0.0036). Further analysis indicated that hsa-miR-210 was positively associated with AFP level, pathological grade, TNM stage, tumor stage and vascular invasion. A total of 168 hsa-miR-210 target genes, which are mainly related to tumor metastasis and tumor signaling pathways, were also predicted in this study.

**Conclusion:** hsa-miR-210 might promote vascular invasion of HCC cells and could be used as a prognostic biomarker.

## Introduction

Hepatocellular carcinoma (HCC) is one of the most common gastro-intestinal cancer and one of the leading causes of cancer-related mortality worldwide (1). HCC is characterized by high malignancy, rapid infiltration, early metastasis and poor therapeutic efficacy (2). In recent years, although the diagnostic and therapeutic approaches for HCC are improved, the prognosis of HCC patients is still very poor. The HCC metastasis and recurrence remarkably reduce the survival rate and patients' quality of life. Therefore, prevention and control of HCC metastasis and recurrence are of great importance for improving the prognosis of HCC patients. With the increasingly deepened understanding of HCC pathogenesis, the microvascular invasion has become a hotspot in HCC research. Former studies suggest that microvascular invasion is a critical risk factor of HCC recurrence (3, 4). Therefore, identification of biomarkers to monitor vascular invasion of HCC will be substantially beneficial for HCC treatment.

MicroRNAs (miRNAs) represent small non-coding RNAs that bind to the 3′ untranslated regions (UTRs) of mRNA transcripts to negatively regulate target gene expression. miRNAs participate in various crucial biological processes such as cell proliferation, apoptosis, development, and differentiation (5, 6). miRNAs can not only be regarded as circulating biomarkers for non-invasive diagnosis and prognosis, but also therapeutic targets in diverse diseases (7, 8). Importantly, it has been demonstrated that miRNAs play important roles in the initiation, progression, metastasis and recurrence of many cancers (9–17).

hsa-miR-210 has been widely studied in many diseases. It has been reported that the role of hsa-miR-210 in HBG2 regulation and induction of HbF makes it valuable for the diagnosis and treatment of Sickle cell disease (18). In addition, multiple studies reveal the important function of hsa-miR-210 in hypoxia and ischemia diseases (19–21). hsa-miR-210 is also dysregulated by hypoxia in many cancers (22–25). Interestingly, hsa-miR-210 participates in the cellular immunity in the tumor microenvironment (26, 27). However, the research of hsa-miR-210 in HCC was insufficient to date. The significance of hsa-miR-210 for the pathogenesis of HCC needs to be revealed.

In this study, we analyzed the dataset GSE6857 using the R package “limma,” and found 7 significantly up-regulated miRNAs in venous metastasis positive HCC tissues. To explore whether these miRNAs are significant for the prognosis of HCC patients, the survival analysis was conducted and results showed that high hsa-miR-210 expression was correlated with a poor outcome of HCC patients. We then verified the expression of hsa-miR-210 in clinical HCC tissues using RT-qPCR, result showed that hsa-miR-210 was significantly high expressed in tissues with portal vein metastasis. In the analysis with clinicopathological variables, hsa-miR-210 showed a close correlation with AFP, pathological grade, TNM stage, tumor stage and vascular invasion. Furthermore, we also identified the hsa-miR-210 target genes and their biological functions. Our data help to understand the crucial role of hsa-miR-210 in HCC.

## Materials and methods

### Study design

**Figure 1:**
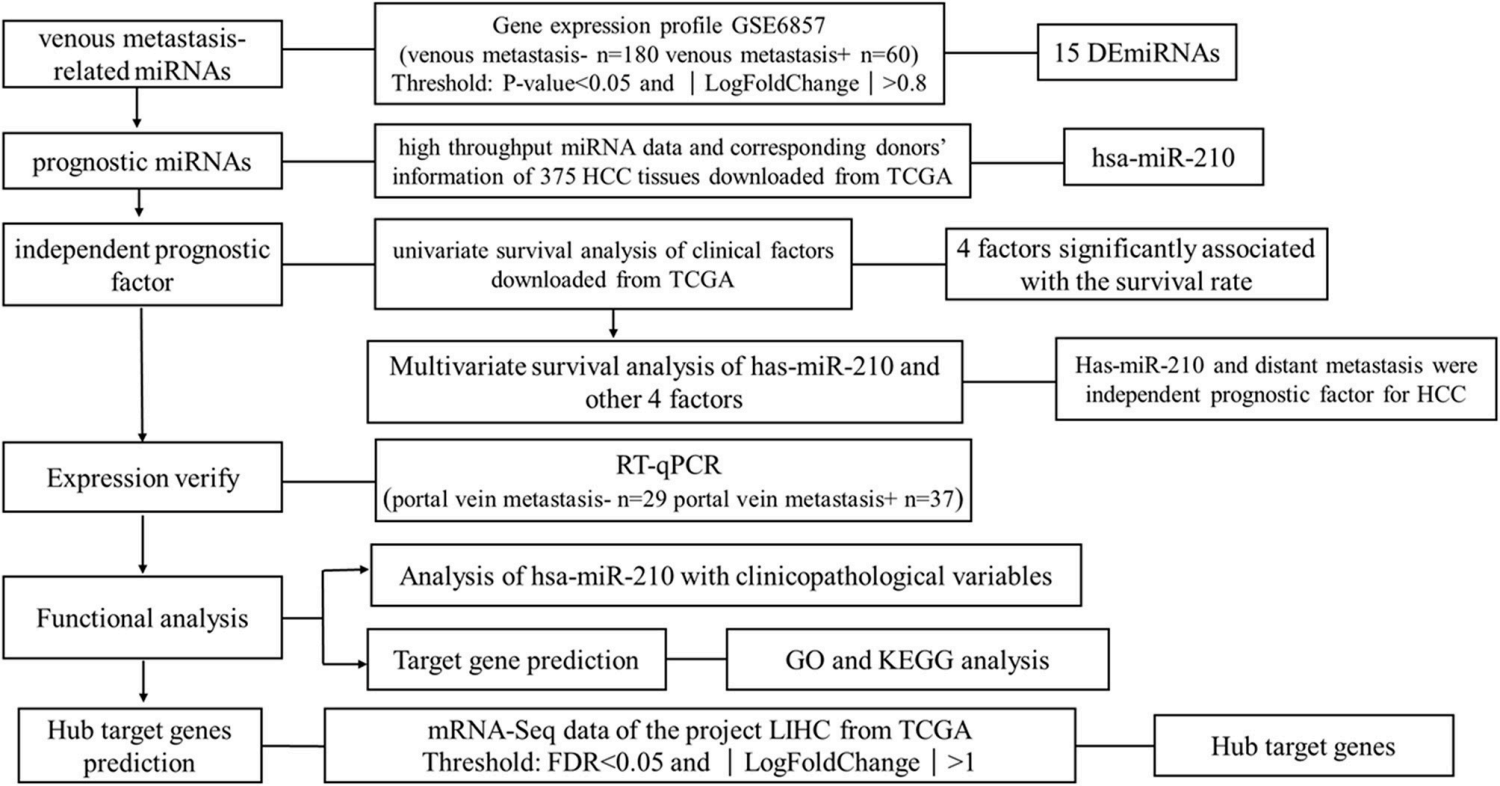
The Overview of the strategy.

We downloaded one dataset from GEO database to screen the venous metastasis-related miRNAs. High throughput miRNA data and clinical information of 375 HCC tissues were downloaded from The Cancer Genome Atlas (TCGA) database to identify miRNAs which are significant for the prognosis of HCC patients. RT-qPCR was conducted to verify the expression of miRNA selected from public database. Other potential functions of selected miRNAs were also analyzed using bioinformatic analysis. The research strategy is embodied in the flowchart (Figure 1).

### Screening of venous metastasis-related miRNAs

Primary data and platform annotation file of dataset GSE6857 (https://www.ncbi.nlm.nih.gov/geo/query/acc.cgi?acc=GSE6857) were downloaded from GEO. The platform of this dataset is OSU-CCC MicroRNA Microarray Version 2.0 (GPL4700) (https://www.ncbi.nlm.nih.gov/geo/query/acc.cgi?acc=GPL4700). The dataset contains the expression profile of 230 miRNAs in 240 paired HCC tissues and adjacent non-tumor liver tissues, 1 lymph node tissue sample of an HCC patient and 1 normal liver tissue. In this study, we chose 180 HCC tissues without venous metastasis and 60 HCC tissues with venous metastasis to conduct the analysis. Significantly differentially expressed miRNAs were selected using R package “limma” (28), while *P* < 0.05 and ∣ Log_2_FoldChange ∣ >0.8 was considered the threshold to judge differentially expressed miRNAs.

### Screening of prognostic miRNAs

The high throughput miRNA data of 375 HCC tissues and corresponding donors' information were downloaded from TCGA (*https://cancergenome.nih.gov/*). Kaplan-Meier survival analysis was conducted to find miRNAs related to the survival of HCC patients. Cox proportional hazards regression analysis was used to determine the variates which are independent prognostic factors in HCC.

### Verifying using clinical samples

#### Sample collection

Aiming to verify the expression of miRNAs selected using public databases, we collected 37 HCC samples with portal vein metastasis and 29 HCC samples without portal vein metastasis. All the clinical specimens were obtained from patients who received liver cancer resection in Zhongnan Hospital of Wuhan University from March of 2018 to October of 2018. Each patient was diagnosed with a histopathological examination and didn't receive any treatment before surgery. Fresh samples were first incubated in RNA stable liquid (RNAlater®) at 4°C for one night and then stored at −80°C for further use. All the work was under approval of Institutional Review Board, and written informed consent was obtained from all participants.

#### Real-time quantitative PCR (RT-qPCR) analysis

Total RNA was isolated using TRIzol reagent (Invitrogen, Life Technologies) and the quality of RNA was assessed by a Thermo Scientific™ Nanodrop 2000 platform (Thermo Fisher Scientific, Waltham, MA, United States). Then reverse transcription was performed using Thermo Scientific RevertAid First Strand cDNA Synthesis Kit (Thermo Fisher Scientific, Waltham, United States). The U6 was used as the endogenous control and was amplified simultaneously with target genes. Designed PCR primers were as follows: U6: Forward, 5′-CTCGCTTCGGCAGCACA-3′ and Reverse, 5′-AACGCTTCACGAATTTGCGT-3′; hsa-miR-210: Forward, 5′-CTGTGCGTGTGACAGC-3′ and Reverse, 5′-GTGCAGGGTCCGAGGT-3′. The reactions started at 95°C for 5 min, followed by 42 cycles of 95°C for 30 s, 61°C for 30 s, and 72°C for 30 s. All experiments were carried out in duplicate for each data point. The relative expression level of hsa-miR-210 was calculated using the comparative Ct method formula 2^−ΔCt^.

### Analysis of the correlations of miRNAs with clinicopathological variables

Clinical characteristics including age, gender, AFP level, pathological grade, TNM stage, tumor stage, lymph node metastasis, distant metastasis, child-pugh score, liver fibrosis, vascular invasion and race of the donors of 375 HCC tissues were downloaded from TCGA. Spearman's rank correlation was employed to analyze the relationship between miRNAs with clinicopathological variables. *P* < 0.05 was considered statistically significant.

### miRNA target prediction and functional analysis

miRNA target genes were predicted using 2 prediction databases including miRanda (Good mirSVR score, Conserved miRNA) (29) (*www.miranda.org*), and TargetScan (30) (http://www.targetscan.org/vert_71/). Only genes that were confirmed by both databases were considered miRNA target genes.

To comprehensively investigate the potential roles of indicated miRNAs in HCC, GO (31) enrichment analysis and KEGG (32) analysis of miRNA target genes were conducted by the online tool DAVID (*https://david.ncifcrf.gov/*, version 6.8). *P* < 0.05 was set as the cutoff.

### Hub target gene prediction

After confirming the miRNA target genes, to identify the chief mRNAs underlying the regulation mechanism of these miRNAs, high-throughput mRNA sequencing data from the LIHC project of TCGA database were used (data downloaded on March 6th, 2018). LIHC project contains 374 human HCC samples and 50 normal liver samples, in which the expression of 60244 genes was detected. Gene annotation file was downloaded from Ensembl (GRCh38) (33) database (*http://www.ensembl.org/index.html*) to analyze the types of these genes. Only protein-coding genes were selected for further analysis. The significantly differentially expressed mRNAs were identified using the R package “edgeR” (34) and “DESeq” (35). Benjamini-Hochberg method was used to control the false discovery rate (FDR). FDR < 0.05 and ∣Log_2_FoldChange∣>1 were set as the threshold to distinguish differentially expressed genes (DEGs). The intersection of mRNAs selected using two methods of analysis were considered as the final DEGs between HCC tissues and normal liver tissues.

Given that miRNAs negatively regulate their target genes, we took the intersection of the down-regulated genes in DEGs with the target genes of those up-regulated miRNAs, while the up-regulated genes in DEGs were compared with the target genes of the down-regulated miRNAs. The mutual ones were considered hub target genes.

### Statistical analysis

Significantly differentially expressed genes in microarray data were analyzed using R package “limma,” and the high throughput miRNA data of TCGA were normalized using Trimmed Mean of M-values (TMM) method by R package “edgeR.” The version of R software was 3.2.5.

Kaplan-Meier survival analysis was conducted for univariate survival analysis, while Cox proportional hazards regression analysis was used for multivariate survival analysis (the analysis method is Forward:LR). The log-rank test was used to determine the difference in survival rates between two or more groups. Spearman's rank correlation was employed to analyze the relationship between miRNAs and clinicopathological variables. Differences between two group of samples were compared using Student's *t*-test or Mann Whitney test. Data were analyzed by GraphPad Prism V.6.00 software (GraphPad Software, San Diego CA, United States) and SPSS 19.0 (SPSS, Inc., Chicago, IL, United States). *P* < 0.05 was considered statistically significant.

## Results

### Venous metastasis-related miRNAs in HCC

To identify the miRNAs critical for venous metastasis in HCC, we analyzed the differentially expressed miRNAs between HCC tissues with or without venous metastasis. Based on the pre-set screening threshold, the expression of 15 miRNAs was notably different between two groups. hsa-miR-301, hsa-let-7e, hsa-let-7b, hsa-miR-31, hsa-miR-210, hsa-miR-371, and hsa-miR-183 were upregulated while hsa-miR-367, hsa-miR-22, hsa-miR-182, hsa-miR-100, hsa-miR-1b-1, hsa-miR-101, hsa-miR-124a-1, and hsa-miR-101 were down-regulated in HCC tissues with venous metastasis, in comparison with those in HCC tissues without venous metastasis (Table 1).

**Table 1:** Significantly differentially expressed miRNAs in venous metastasis positive HCC tissues.

**miRNA**	**Screening dataset**		
	**Log FC**	**Adj *P*-value**	**Type**
hsa-miR-301	0.842786	0.000267078	Up
hsa-let-7e	0.847988	9.04E-11	Up
hsa-let-7b	0.850236	2.94E-12	Up
hsa-miR-31	0.853136	5.34E-07	Up
hsa-miR-210	0.868472	1.68E-06	Up
hsa-miR-371	0.991324	1.92E-10	Up
hsa-miR-183	1.027867	1.14E-05	Up
hsa-miR-367	−1.08021	1.81E-06	Down
hsa-miR-22	−0.9947	0.00055601	Down
hsa-miR-182	−0.94816	0.00023209	Down
hsa-miR-100	−0.94307	9.38E-07	Down
hsa-miR-1b-1	−0.87813	0.00015521	Down
hsa-miR-101	−0.84328	0.000209509	Down
hsa-miR-124a-1	−0.81885	2.27E-08	Down
hsa-miR-101	−0.80452	9.15E-10	Down

### hsa-miR-210 is associated with the prognosis of HCC patients

**Figure 2:**
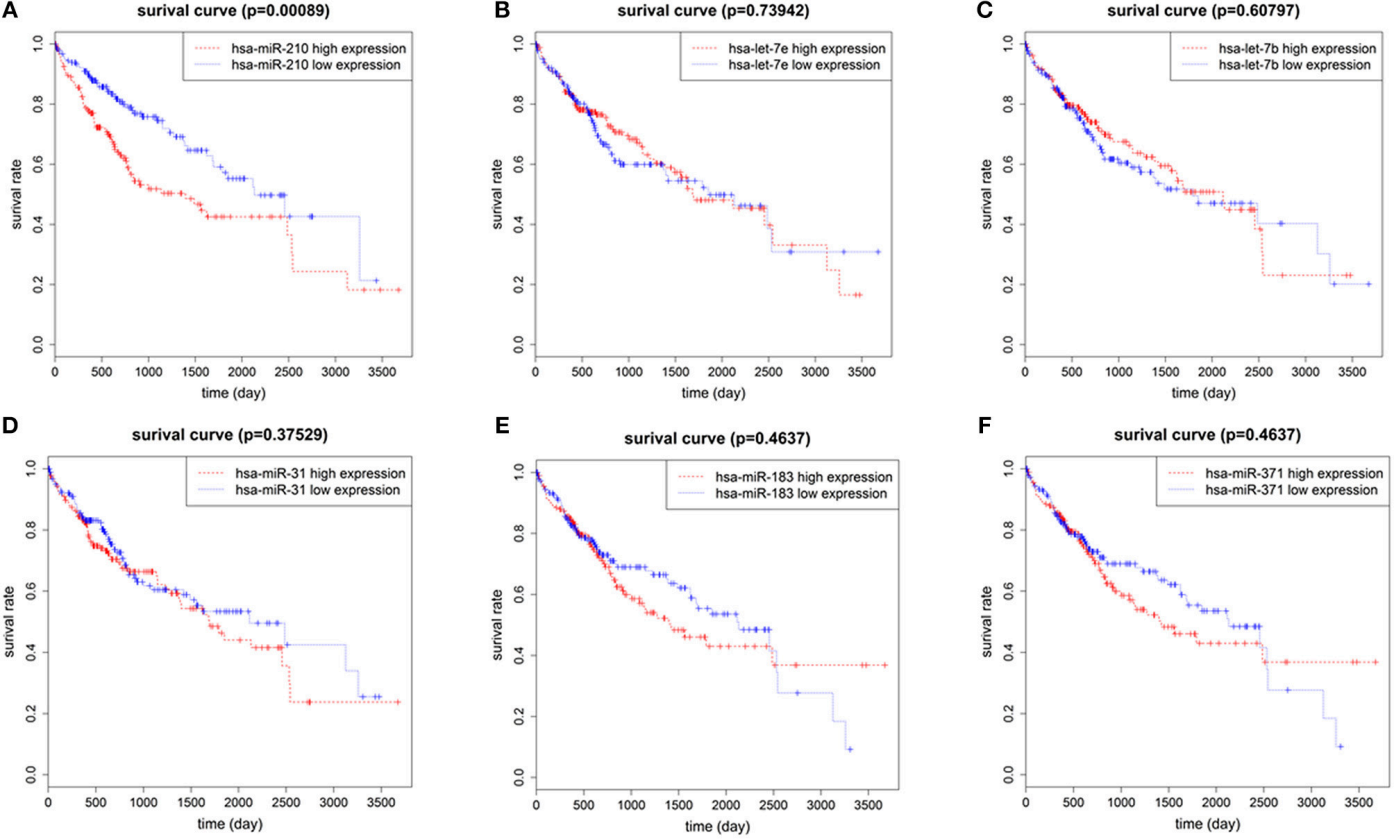
The correlation of 6 up-regulated miRNAs with overall survival of HCC patients. Survival analysis of hsa-miR-210 **(A)**; hsa-let-7e **(B)**; hsa-let-7b **(C)**; hsa-miR-31 **(D)**; hsa-miR-183 **(E)**; hsa-miR-371 **(F)**.

To identify the miRNAs associated with the prognosis of HCC patients, we used normalized expression of 7 up-regulated miRNAs and the survival data (overall survival time and living state) of 375 HCC patients in TCGA. We found that the overall survival rate of patients with high hsa-miR-210 expression was significantly lower than the survival rate of patients with low hsa-miR-210 expression. Other miRNAs such as hsa-let-7e, hsa-let-7b, hsa-miR-31, hsa-miR-371 and hsa-miR-183 had no significant influence on the survival rate of HCC patients (Figure 2). We didn't analyze hsa-miR-301 because we only found the expression of hsa-miR-301a and hsa-miR-301b in TCGA. According to miRNA nomenclature (36, 37), hsa-miR-301a and hsa-miR-301b are highly homologous to hsa-miR-301. They may have the same molecular function with hsa-miR-301, but there exists difference between the expression.

### hsa-miR-210 is an independent prognostic factor for HCC

A multivariate survival analysis was conducted to explore whether hsa-miR-210 is an independent prognostic factor for HCC. At first, a univariate survival analysis was conducted. Clinical characteristics including age, gender, AFP level, pathological grade, TNM stage, tumor stage, lymph-node metastasis, distant metastasis, child-pugh score, liver fibrosis, vascular invasion and race were subject to Kaplan-Meier analysis to identify the factors influencing the survival rate of HCC patients. As a result, AFP, TNM stage, tumor stage and distant metastasis were significantly associated with the survival rate of HCC patients (Table 2 and Figure S1).

**Table 2:** Univariate analysis of factors affecting overall survival of HCC patients.

**Variable**	**Group**	**Case number**	***P***
Age	< 50	71	0.173
	≥50	301	
Gender	Male	253	0.309
	Female	119	
AFP	< 20	150	**0.019**
	≥20	130	
Grade	1	55	0.858
	2	176	
	3	123	
	4	13	
TNM stage	I	173	**0.000**
	II	85	
	III	85	
	IV	5	
Tumor stage	T1	183	**0.000**
	T2	93	
	T3	80	
	T4	13	
Lymph-node metastasis	N0	254	0.317
	N1	4	
Distant metastasis	M0	269	**0.01**
	M1	4	
Child-pugh	A	219	0.357
	B	21	
	C	1	
Liver fibrosis	No	75	0.316
	Yes	139	
Vascular invasion	No	206	0.162
	Yes	110	
Race	Asian	160	0.129
	Others	202	

*Note: The statistically significant values are in bold*.

Cox proportional hazards regression analysis was then conducted to carry out the multivariate survival analysis. Five variates, which were demonstrated to be significant in the univariate analysis, entered the Cox hazard model to test their independent impact on HCC. Based on this analysis, high hsa-miR-210 level was identified as an independent prognostic factor for HCC (HR 2.627; 95% CI 1.516–4.551; *p* = 0.001). Another variate, distant metastasis, was a potential independent prognostic factor for HCC (HR 5.146; 95% CI 1.580–16.760; *p* = 0.007). However, other variates including AFP, TNM stage and tumor stage had no significant influence on the overall survival time (Table 3).

**Table 3:** Cox proportional hazards regression analysis of factors affecting overall survival of HCC patients.

**Variable**	**B**	**SE**	**Sig**.	**HR**	**95% CI**
hsa-miR-210	0.966	0.280	0.001	2.627	1.516–4.551
Distant metastasis	1.638	0.602	0.007	5.146	1.580–16.760

### hsa-miR-210 up-regulate in portal vein metastasis HCC samples

**Figure 3:**
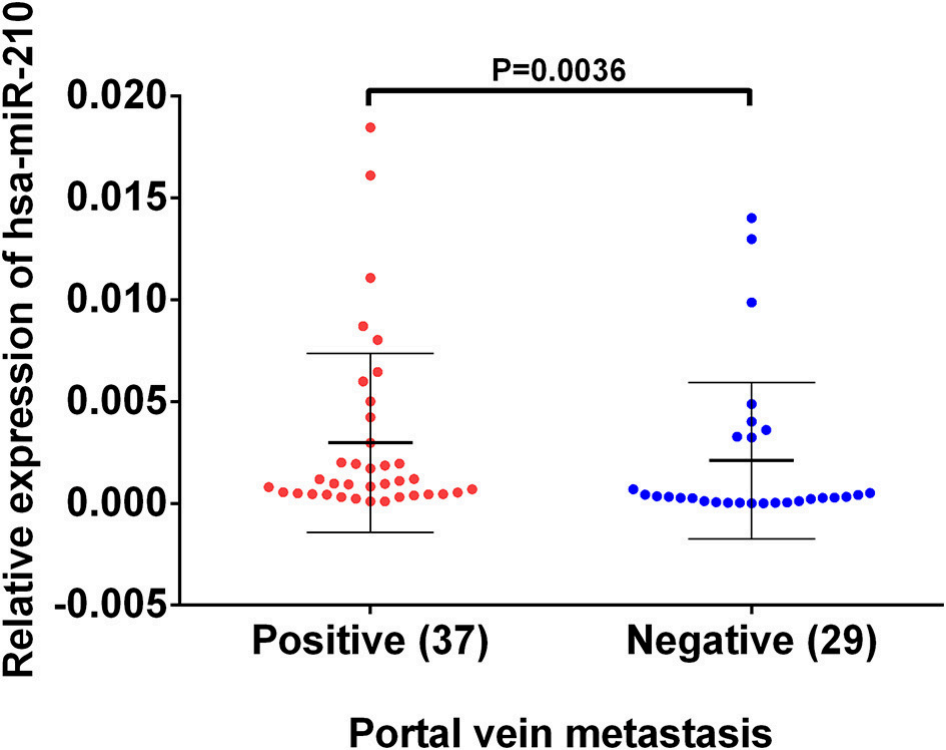
The expression of hsa-miR-210 in HCC samples with positive and negative portal vein metastasis. The relative expression was determined using RT-qPCR. hsa-miR-210 levels in the portal vein metastasis positive tissues were higher than those in portal vein metastasis negative tissues.

Based on the previous analysis using public database, we found high hsa-miR-210 expression in venous metastasis HCC tissues. Aiming to verify this expression trend, we conducted RT-qPCR assay on 37 HCC samples with portal vein metastasis and 29 HCC samples without venous metastasis. The results showed that the mean value of hsa-miR-210 in portal vein metastasis positive tissues and negative HCC tissues were 0.00098 vs. 0.00032, respectively (*P* = 0.0036). The finding suggests that expression of hsa-miR-210 was significantly higher in HCC samples with portal vein metastasis (Figure 3).

### The correlation of hsa-miR-210 with HCC clinicopathological variables

To clarify the relationship between HCC and hsa-miR-210, we analyzed the correlation of hsa-miR-210 with HCC clinicopathological variables.

Notably, our analysis showed that hsa-miR-210 expression was positively associated with vascular invasion. What's more, high expression of has-miR-210 portends high AFP level, pathological grade, TNM stage and tumor stage, which verified the essential role of hsa-miR-210 in influencing the degree of tumor malignant. But its expression had no significant correlation with age, gender, Lymph-node metastasis, distant metastasis, child-pugh, liver fibrosis and race (Table 4 and Figure S2).

**Table 4:** The relationship between hsa-mir-210 and the clinicopathological variables of HCC.

	**Group**	**Case**	**hsa-miR-210**	
			***R***	***p***
Age	< 50	71	0.072	0.164
	≥50	300		
Gender	Male	253	0.093	0.074
	Female	119		
AFP	< 20	150	0.095	0.112
	≥20	131		
Grade	1	55	**0.144**	**0.006**
	2–4	313		
TNM stage	I–II	258	**0.197**	**0.00**
	III–IV	90		
Tumor stage	T0–T1	182	**0.162**	**0.002**
	T2–T4	187		
Lymph-node metastasis	N0	254	0.061	0.332
	N1	4		
Distant metastasis	M0	269	−0.005	0.934
	M1	4		
Child-pugh	A	220	0.028	0.67
	B–C	22		
Liver fibrosis	No	75	−0.087	0.206
	Yes	140		
Vascular invasion	No	206	**0.135**	**0.016**
	Yes	111		
Race	Asian	160	−0.024	0.647
	Others	202		

*Note: The statistically significant values are in bold*.

### Target gene prediction and functional analysis

To provide deep insight into the role of hsa-miR-210 in the HCC initiation and progression, hsa-miR-210 target genes were predicted by two prediction databases. 684 target genes and 4,059 target genes were found from miRanda database and TargetScan database, respectively. Among them, a total of 168 common genes were identified as hsa-miR-210 target genes (Table S1). In particular, 15 predicted target genes have been indicated to be regulated by hsa-miR-210 in former studies.

**Figure 4:**
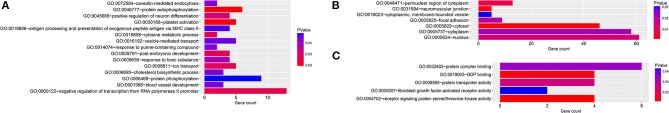
GO analysis of hsa-miR-210 target genes. GO enrichment of target genes in biological process ontology **(A)**, cellular component ontology **(B)** and molecular function ontology **(C)**.

**Figure 5:**
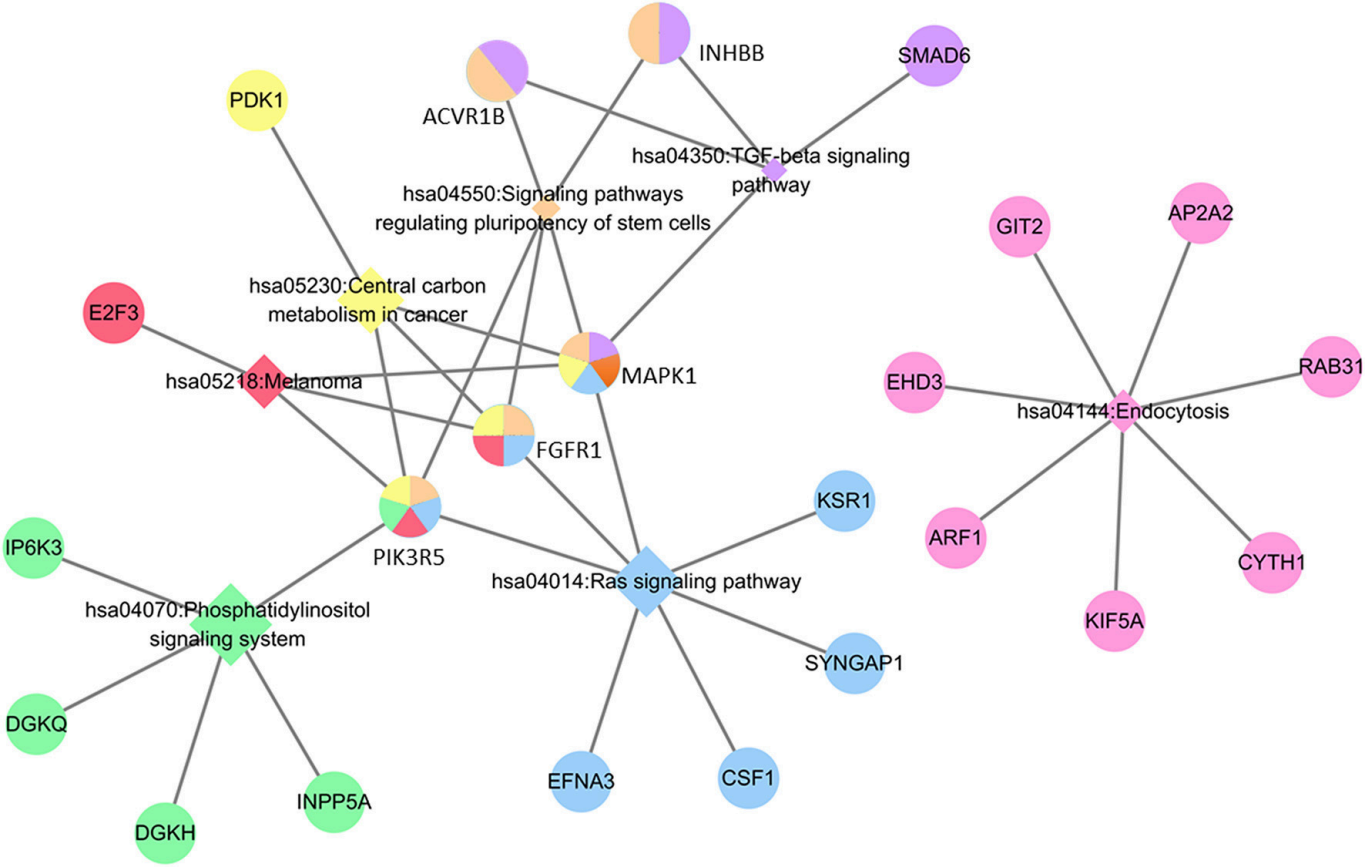
KEGG analysis of hsa-miR-210 target genes. The circle represents target genes; the diamonds represent signaling pathways. Circles with the same color with diamonds indicate the target genes participating in the corresponding signaling pathways. The relationship between each signaling pathway and each gene is represented by a gray line. The network was generated by Cytoscape_3.5.1.

Regarding the function and pathway enrichment analysis of the target genes, the GO analysis of the hsa-miR-210 target genes was summarized in Figure 4. The significantly enriched items in each domain were demonstrated according to their *p*-values. hsa-miR-210 target genes were mainly enriched in the biological processes including blood vessel development, protein autophosphorylation, platelet activation and negative regulation of transcription from RNA polymerase II promoter. The products of these target genes are involved in various functions including receptor signaling protein serine/threonine kinase activity, fibroblast growth factor-activated receptor activity and protein complex binding. Moreover, they are localized in the nucleus or cytoplasm. KEGG analysis revealed that hsa-miR-210 target genes mainly participate in cancer-related signaling pathways like Ras signaling, TGF-β signaling, phosphatidylinositol signaling, and central carbon metabolism. They were also involved in the signaling pathways regulating stem cell pluripotency (Figure 5). Notably, INHBB, ACVR1B, MAPK1, FGFR1, and PIK3R5 were involved in multiple pathways.

### Hub target genes of hsa-miR-210

**Figure 6:**
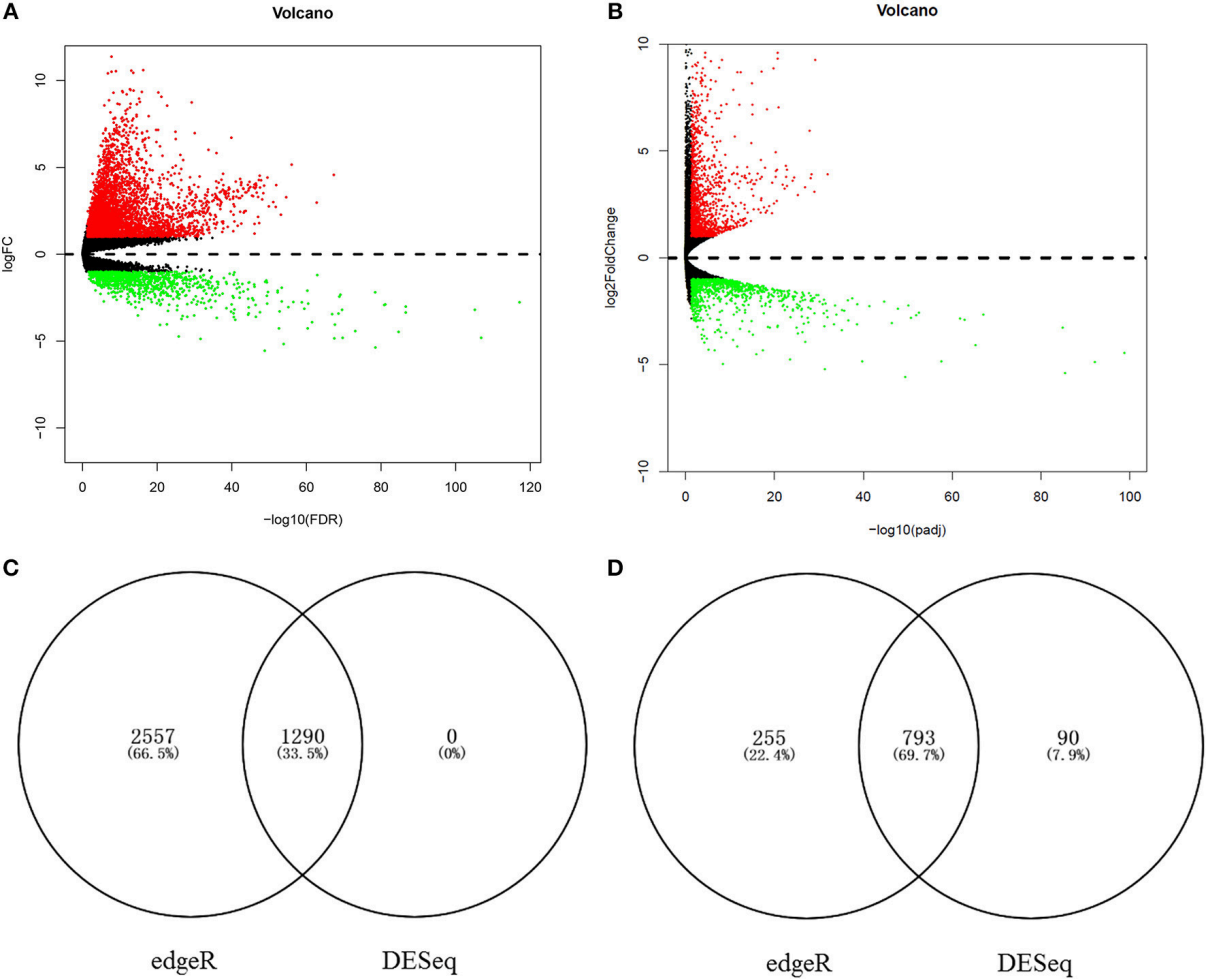
The aberrantly expressed mRNAs in HCC tissues. Aberrantly expressed miRNAs calculated by edgeR R. 3847 up-regulated and 1048 down-regulated miRNAs were found **(A)**; Aberrantly expressed miRNAs calculated by DESeq R. 1290 up-regulated and 883 down-regulated miRNAs were found **(B)**. Red dots indicate up-regulated miRNAs and green dots indicate down-regulated miRNAs. Black dots show the miRNAs expression with |log_2_FC| < 1. The X-axis represents an adjusted FDR value and the Y-axis represents the log_2_FC value. The volcano plot was generated by the ggplot2 package of R language. The intersection of up-regulated mRNAs obtained using edgeR R and DESeq R **(C)**. The intersection of down-regulated mRNAs obtained using edgeR R and DESeq R **(D)**.

To identify the chief mRNAs that participate in the regulation of hsa-miR-210, the high-throughput mRNA sequencing data from TCGA was introduced. The differential expression analysis using R package “edgeR” revealed 4895 differentially expressed genes. Among them, 3847 genes were up-regulated while 1048 genes were down-regulated (Figure 6A). 2173 mRNAs were obtained using differential expression analysis conducted by R package “DESeq”, thereinto, 1290 genes were up-regulated while 883 genes were down-regulated (Figure 6B). After taking the intersection of the result of two analysis, we got 1290 mRNAs up regulated in HCC tissues (Figure 6C) and 793 mRNAs significantly down regulated (Figure 6D).

The intersection between the differentially expressed genes and the predicted hsa-miR-210 target genes was set according to the negative correlation between miRNAs and their target genes. hsa-miR-210, which was up-regulated in HCC, significantly down-regulated 7 target genes (JDP2, SAA1, CR1, SHMT1, KMO, NOL4, and EHD3) in HCC tissues.

## Discussion

Metastasis is one of the main malignant characters of cancers. It is responsible for up to 90% of cancer-related mortality (38). Metastasis involves a series of complex cascades including cancer cell invasion into the surrounding tissue, infiltration across blood vessels and lymphatic vessels, migration to distant tissues and organs, colonization in distant tissues and organs (39). The vascular invasion has been proven to be an important indicator of HCC recurrence after hepatectomy and liver resection (40). Early detection and treatment of vascular invasion could help reduce the recurrence and prolong the survival of patients.

As a hypoxia-inducible factor, hsa-miR-210 has been confirmed by multiple studies to play a crucial role in tumor proliferation, metastasis and invasion. High hsa-miR-210 expression promotes the metastasis and indicates poor prognosis of gastric cancer and colorectal cancer (41, 42). hsa-miR-210 also plays a central role in tumor growth through participating in the induction of cell senescence, regulation of the recruitment of inflammatory cells, formation of tumor blood vessels, and generation of high-energy metabolites to maintain (43). Moreover, hsa-mir-210 is also used to assess drug susceptibility or as a therapeutic target (44).

In this study, through analyzing the miRNA expression profile of HCC tissues with or without venous metastasis, we found that hsa-miR-210 was significantly increased in venous metastasis positive samples. Moreover, high hsa-miR-210 expression was closely correlated with poor HCC outcome in both univariate and multivariate survival analysis. Additionally, hsa-miR-210 expression was positively correlated with AFP, pathological grade, TNM stage, tumor stage and vascular invasion, suggesting that hsa-miR-210 indicate the extent of HCC malignancy. It has been proposed that hsa-miR-210 promotes the metastasis of hypoxic HCC cells by directly targeting the metastasis suppressor vacuole membrane protein 1 (VMP1) (45). Meanwhile, it was reported that high hsa-miR-210 expression is closely associated with the poor prognosis including short tumor-free survival time and low survival rate (46). Previous studies also demonstrated that hsa-miR-210 promotes HCC angiogenesis through downregulating FGFRL1. Additionally, an *in vitro* research confirmed that high hsa-mir-210 expression promotes tumor cell proliferation, inhibits apoptosis and reduces the radiosensitivity of HCC cells in low oxygen environment (47). To our knowledge, we are the first to unveil that hsa-miR-210 promotes vascular invasion in HCC through bioinformatics analysis.

To explore the biological function of hsa-miR-210 and the signaling pathways it regulates, we used two miRNA target gene prediction databases to obtain hsa-miR-210 target genes. Among these genes, the expression of BDNF, ZNF462, GIT2, GPD1L, ISCU, NFIC, FGFRL1, MNT, EFNA3, and E2F3 have been shown to be regulated by hsa-miR-210 in previous studies (48–56). Other target genes which haven't been validated by experiments might be clues for exploring new biological functions of hsa-miR-210 in HCC. In GO and KEGG analysis, we found that the biological processes and molecular functions of these target genes were closely related to malignancy and metastasis of tumors. Additionally, hsa-miR-210 was involved in many tumorigenesis-related signaling pathways, such as Ras signaling (57), TGF-β signaling (58), and phosphatidylinositol signaling (59, 60). Particularly, the regulatory effects of hsa-miR-210 on TGF-β signaling and phosphatidylinositol signaling have been confirmed previously (61, 62). hsa-miR-210 also influences the signaling pathways involved in stem cell pluripotency and central carbon metabolism. In our research, we found that MAPK1, FGFR1, and PIK3R5 participate in more than 4 important cancer-related signaling pathways. Nonetheless, no study has shown the effect of hsa-miR-210 on their expression. Therefore, investigation on these genes will facilitate a deeper understanding of hsa-miR-210 in HCC. In addition, the molecular mechanisms by which hsa-mir-210 promotes HCC vascular invasion requires further investigation, and so is the case for other candidate target genes of hsa-miR-210.

In conclusion, this study demonstrates that hsa-mir-210 promote venous metastasis in HCC. hsa-mir-210 may be a promising biomarker for evaluating the prognosis of HCC patients.

## Author contributions

All authors listed have made a substantial, direct and intellectual contribution to the work, and approved it for publication.

### Conflict of interest statement

The authors declare that the research was conducted in the absence of any commercial or financial relationships that could be construed as a potential conflict of interest.
