# Supplementary material for: Up-Regulation of hsa-miR-210 Promotes Venous Metastasis and Predicts Poor Prognosis in Hepatocellular Carcinoma
Source: Front Oncol. 2018 Dec 3;8:569. doi: 10.3389/fonc.2018.00569 (PMC6287006; doi:10.3389/fonc.2018.00569)
Supplement: Supplementary file 1 [file Data_Sheet_1.docx]

Supplementary Material

**Up-regulation of hsa-miR-210 Promotes Venous Metastasis and Predicts Poor Prognosis in Hepatocellular Carcinoma**

**Jia Ji ^#^, Yuan Rong^#^, Chang-Liang Luo, Shuo Li, Xiang Jiang, Hong Weng, Hao Chen, Wu-Wen Zhang, Wen Xie^*^, Fu-Bing Wang^*^**

*** Correspondence:**

^*^Corresponding author: Fu-Bing Wang, Department of Laboratory Medicine, Zhongnan Hospital of Wuhan University, No 169 Donghu Road, Wuchang District, Wuhan 430071, P.R. China. Email address: wfb20042002@sina.com; Tel: +86-27-67813517; Fax: +86-27-67813128; and Wen Xie, [whxw007@126.com](mailto:whxw007@126.com).

# Supplementary Figures and Tables

## Supplementary Figures

**
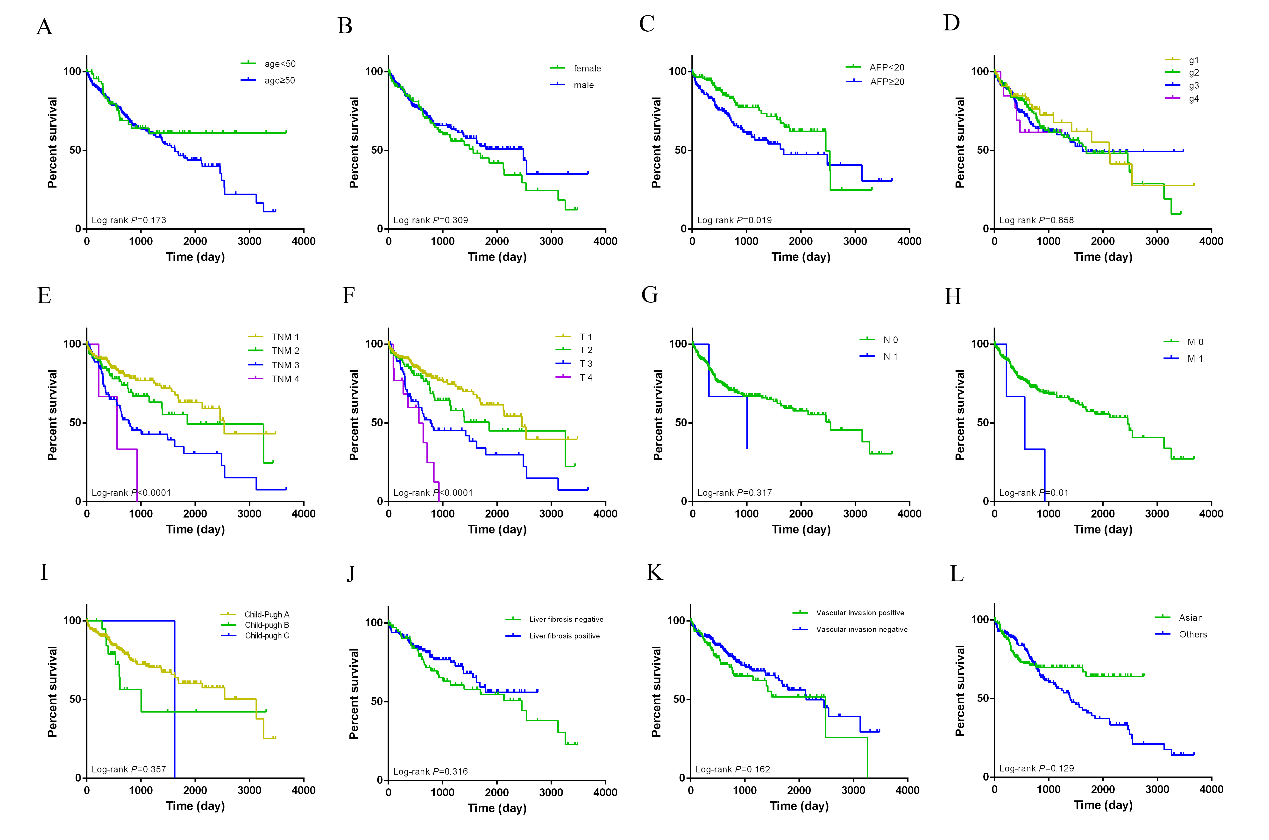
**

**Figure S1. Correlation of 12 Clinical characteristics with overall survival of HCC patients**

**Notes:** Survival analysis of age(A); gender(B); AFP level(C); pathological grade(D); TNM stage(E); tumor stage(F); lymph-node metastasis(G); distant metastasis(H); child-pugh score(I); liver fibrosis(J); vascular invasion(K); race(L).

**
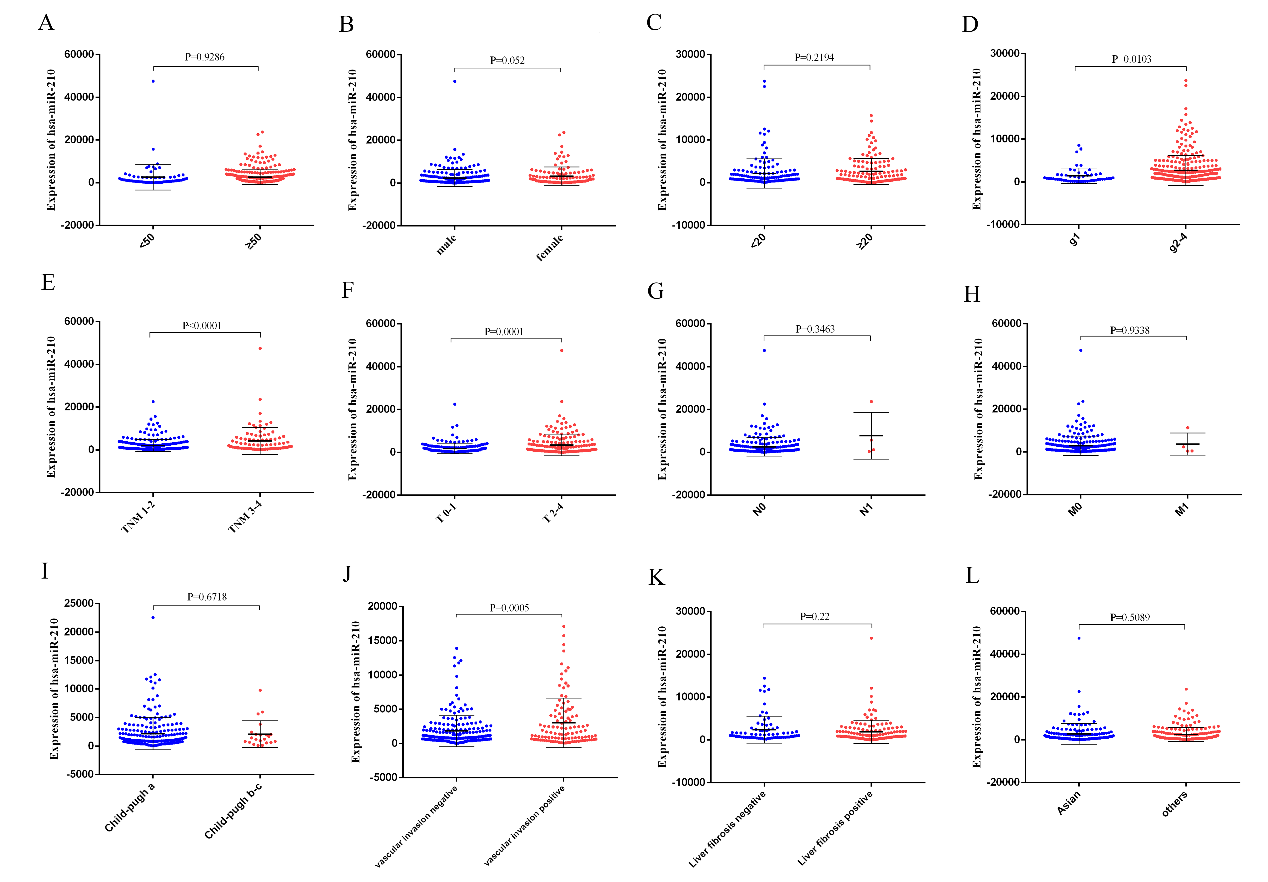
**

**Figure S2. Expression of hsa-miR-210 in different subgroups**

**Note:** Expression of hsa-miR-210 in subgroups of age(A); gender(B); AFP level(C); pathological grade(D); TNM stage(E); tumor stage(F); lymph-node metastasis(G); distant metastasis(H); child-pugh score(I); liver fibrosis(J); vascular invasion(K); race(L).

## Supplementary Table

**Table S1.** Target genes of hsa-miR-210

| miRNA | target gene |
| --- | --- |
| hsa-miR-210 | ACTN4，ACVR1B(1)，ADAMTSL1，ALDH5A1，ANKHD1-EIF4EBP3，ANTXR1，AP2A2，AQP4，ARC，ARF1，ARHGEF17，ATF7IP，ATG7(2)，ATP5SL，BAZ2B，BDNF(3)，BEND4，BZRAP1，C1QTNF3，CCDC88B，CDHR3，CDK13，CENPP，CORO2B，CORO7，COX19，CR1，CSF1，CSMD2，CYGB，CYTH1，DAK，DGKH，DGKQ，DHCR7，DNMT3B(4)，E2F3(5)，EFNA3(5)，EHD3，ENAM，EPHA7，F7，FAM124B，FAM27E3，FAM73B，FAM89B，FCHSD2，FGD4，FGFR1，FGFRL1(6)，FLNC，FTSJ2，GABRB1，GDE1，GIT2(5)，GPD1L(3)，GPR123，GPR17，GPR97，HCFC2，HDAC4，HMBOX1，HMGCS1，INF2，INHBB，INPP5A，INSIG1，IP6K3，IRF2，ISCU(7)，ITGB3BP，ITPRIP，JDP2，KDM4A，KIAA1161，KIF5A，KLHL29，KMO，KPNA1，KSR1，LAIR1，LOXL4，LRFN1，MAML3，MAN2A2，MAPK1，MAPT，MBTD1，MCM8，MED20，MGRN1，MID1IP1，MLC1，MLH3，MNT(5)，MRPL36，MRTO4，MRVI1，NDRG4，NDUFA10，NDUFA4，NEUROD2(8)，NFIC(9)，NLRC5，NOL12，NOL4，NPLOC4，OSBPL1A，PDK1(10)，PGAM5，PIK3R5，PLEKHA7，POU2F1，POU3F2，PPM1M，PRMT1，PROK1，PSEN1，PTPRD，RAB31，RAB3B，RAB9B，RBM3，RHBG，RSU1，RTP1，SAA1，SCARA3，SDF2，SHMT1，SIN3B，SLC25A26，SLC2A4RG，SLC6A1，SLC8A3，SMAD6，SMG5，SORBS3，SP140L，ST3GAL3，STARD3，STK24，STK25，SYNGAP1，TANC2，TAOK3，TBC1D30，TCTN1，TET2，TIGD5，TMEM135，TMEM30B，TMEM40，TNPO3，TOMM40L，TPD52L2，TRIM41，ULK1，USP6NL，WDFY2，WHSC1，WHSC1L1，ZFAND3，ZNF446，ZNF462(5)，ZNF626，ZNF697，ZNF862 |

**Reference**

1. Mizuno Y, Tokuzawa Y, Ninomiya Y, Yagi K, Yatsuka-Kanesaki Y, Suda T, et al. miR-210 promotes osteoblastic differentiation through inhibition of AcvR1b. *FEBS letters* (2009) 583(13):2263-8. Epub 2009/06/13. doi: 10.1016/j.febslet.2009.06.006. PubMed PMID: 19520079.

2. Wang C, Zhang ZZ, Yang W, Ouyang ZH, Xue JB, Li XL, et al. MiR-210 facilitates ECM degradation by suppressing autophagy via silencing of ATG7 in human degenerated NP cells. *Biomedicine & pharmacotherapy = Biomedecine & pharmacotherapie* (2017) 93:470-9. Epub 2017/07/02. doi: 10.1016/j.biopha.2017.06.048. PubMed PMID: 28667916.

3. Fasanaro P, Greco S, Lorenzi M, Pescatori M, Brioschi M, Kulshreshtha R, et al. An integrated approach for experimental target identification of hypoxia-induced miR-210. *The Journal of biological chemistry* (2009) 284(50):35134-43. Epub 2009/10/15. doi: 10.1074/jbc.M109.052779. PubMed PMID: 19826008; PubMed Central PMCID: PMCPMC2787374.

4. Xiong L, Wang F, Huang X, Liu ZH, Zhao T, Wu LY, et al. DNA demethylation regulates the expression of miR-210 in neural progenitor cells subjected to hypoxia. *The FEBS journal* (2012) 279(23):4318-26. Epub 2012/10/09. doi: 10.1111/febs.12021. PubMed PMID: 23039253.

5. Chen WY, Liu WJ, Zhao YP, Zhou L, Zhang TP, Chen G, et al. Induction, modulation and potential targets of miR-210 in pancreatic cancer cells. *Hepatobiliary & pancreatic diseases international : HBPD INT* (2012) 11(3):319-24. Epub 2012/06/08. PubMed PMID: 22672828.

6. Tsuchiya S, Fujiwara T, Sato F, Shimada Y, Tanaka E, Sakai Y, et al. MicroRNA-210 regulates cancer cell proliferation through targeting fibroblast growth factor receptor-like 1 (FGFRL1). *The Journal of biological chemistry* (2011) 286(1):420-8. Epub 2010/11/04. doi: 10.1074/jbc.M110.170852. PubMed PMID: 21044961; PubMed Central PMCID: PMCPMC3013001.

7. Favaro E, Ramachandran A, McCormick R, Gee H, Blancher C, Crosby M, et al. MicroRNA-210 regulates mitochondrial free radical response to hypoxia and krebs cycle in cancer cells by targeting iron sulfur cluster protein ISCU. *PLoS One* (2010) 5(4):e10345. Epub 2010/05/04. doi: 10.1371/journal.pone.0010345. PubMed PMID: 20436681; PubMed Central PMCID: PMCPMC2859946.

8. Agrawal R, Garg A, Benny Malgulwar P, Sharma V, Sarkar C, Kulshreshtha R. p53 and miR-210 regulated NeuroD2, a neuronal basic helix-loop-helix transcription factor, is downregulated in glioblastoma patients and functions as a tumor suppressor under hypoxic microenvironment. *Int J Cancer* (2018) 142(9):1817-28. Epub 2017/12/12. doi: 10.1002/ijc.31209. PubMed PMID: 29226333.

9. Zhang H, Mai Q, Chen J. MicroRNA-210 is increased and it is required for dedifferentiation of osteosarcoma cell line. *Cell biology international* (2017) 41(3):267-75. Epub 2016/12/30. doi: 10.1002/cbin.10721. PubMed PMID: 28032372.

10. Li Y, Yang C, Zhang L, Yang P. MicroRNA-210 induces endothelial cell apoptosis by directly targeting PDK1 in the setting of atherosclerosis. *Cellular & molecular biology letters* (2017) 22:3. Epub 2017/05/26. doi: 10.1186/s11658-017-0033-5. PubMed PMID: 28536634; PubMed Central PMCID: PMCPMC5415835.
